# Reality bites: An analysis of corona deniers in Germany over time

**DOI:** 10.3389/fsoc.2022.974972

**Published:** 2022-11-04

**Authors:** Jan Eichhorn, Tobias Spöri, Jan Delhey, Franziska Deutsch, Georgi Dragolov

**Affiliations:** ^1^Social Policy, University of Edinburgh, Edinburgh, United Kingdom; ^2^d|part, Berlin, Germany; ^3^Soziologie, Otto von Guericke University Magdeburg, Magdeburg, Saxony-Anhalt, Germany; ^4^Social Sciences and Humanities, Jacobs University Bremen, Bremen, Germany

**Keywords:** conspiracy theory, COVID-19, Germany, political attitudes, values

## Abstract

The COVID-19 pandemic resulted in unprecedented government interventions in many people's lives. Opposition to these measures was not only based on policy disagreements but for some founded in an outright denial of basic facts surrounding the pandemic, challenging social cohesion. Conspiracy beliefs have been prolific within various protest groups and require attention, as such attitudes have been shown to be associated with lower rule compliance. Several studies have shown that the characteristics linked to holding COVID-19 conspiracy beliefs are complex and manifold; however, those insights usually rest on cross-sectional studies only. We have less knowledge on whether these cross-sectional correlates also reveal which parts of the population have been newly convinced by conspiracy theories or have dropped their support for them as the pandemic evolved. Using a unique panel data set from Germany, this paper explores a wide range of characteristics and compares the insights gained from cross-sectional associations on the one hand and links to the ways in which people change their views on the other hand. The findings show that cross-sectional analyses miss out on nuanced differences between different groups of temporary and more consistent conspiracy supporters. Specifically, this paper identifies major differences in the profiles of people who have been denying COVID-19 consistently compared to those who changed their minds on the question and those who assessed the reality correctly throughout. In doing so, socio-political and perception-based dimensions are differentiated and distinctions between respondents from East and West Germany explored.

## Introduction

In the social sciences, social cohesion is widely seen as an important resource for collectives, especially in times of crisis (Townshend et al., 2015). While being a multi-facetted concept, group members' orientation toward the common good is often considered to be one of the key ingredients of social cohesion (cf. Dragolov et al., 2016). Such a civic and solidary orientation, however, requires a basic understanding within the citizenry about what the common good actually is and in which way it is challenged. While such a collective consciousness, at least in modern-pluralistic societies, does not necessarily extend to moral values (cf. Schiefer and van der Noll, 2017), it certainly does extend to perceptions of social realities in the sense of non-refutable facts. Without a shared understanding of reality, societies will find it more difficult to respond to crises and threats.

Without doubt, the COVID-19 pandemic constitutes a severe threat. The infectiousness and transmissibility of the virus meant that individual action was not sufficient to mitigate its proliferation. Collective action was required to reduce the amount of human interaction at a large scale while protective instruments, such as vaccines and new medicines, could be developed. The response by governments was unprecedented for most people: Extensive mandates resulted in the restriction of personal freedoms at a scale unseen in peacetime. The curtailment of businesses, education, transport, and even the option to leave one's own home and meet others in times of lockdowns represented the most extensive state intervention into people's lives, heavily disrupting life as-we-know it.

It quickly became clear that a resilient collective consciousness necessary to jointly face the pandemic existed in large parts of the population, but not in all: While some questioned the scope and nature of measures implemented, a significant minority of people rejected that the COVID-19 pandemic was actually real. These Corona skeptics or Corona deniers stipulated that the pandemic itself was fabricated. Crucially, such denial had profound behavioral consequences: People who did not believe in the scientifically established facts that a pandemic was ongoing were much less likely to adhere to the protective rules such as mask wearing and social distancing (Allington et al., 2021; Pummerer, 2021) or—once it became available—to get vaccinated (Pivetti et al., 2021). In many countries, Corona deniers joined together in social movements, which operated in Germany, our country of interest, under the name “Querdenker”.

Therefore, understanding how widespread COVID-19 conspiracy theories are and who subscribes to them is important for the development of strategies to engage with people reluctant to comply with public health measures. While a number of studies have provided valuable insights on these issues (for a review, see van van Mulukom et al., 2022), most of them are cross-sectional: They can inform who is more likely to hold conspiracy beliefs at a given point in time, but not how stable corona denial is within individuals over the course of the pandemic. Were those who supported conspiracy beliefs at the start of the pandemic also the same people who held these views later on? Did their socio-political and attitudinal profile change? A *longitudinal* perspective is essential to answer questions like these—and to identify groups where pandemic conspiracy beliefs have become deeply engrained. Considering such dynamics is important: When threat perceptions of conspiracy believers and the population majority develop in an oppositional way, conspiracy beliefs may become even further entrenched (van Prooijen, 2020). Ultimately, this results in the group of conspiracy believers becoming further distanced from the rest of society.

In this paper, we analyze data from a unique panel study of the German population that allows us to investigate those questions. The data stem from an online survey conducted of a sample of people in Germany aged 16 and older that is close to representative of the German population in key demographic and socio-economic parameters. Over 2,000 respondents were interviewed at the start of the pandemic in April and May 2020 and then re-invited to participate in a follow-up survey in February and March 2021 after experiencing the first lockdown, an easing of restrictions, and entering a second lockdown. The data allow us (1) to examine how many individuals held pandemic-related conspiracy beliefs at both or either points of time, and (2) to investigate what socio-demographic and attitudinal profile characterizes temporary (both former and new) and consistent pandemic deniers.

## Conceptual considerations and review of findings

According to Douglas et al. (2019, p. 4), conspiracy theories “are attempts to explain the ultimate causes of significant social and political events and circumstances with claims of secret plots by two or more powerful actors”. A conspiracy belief, then, is the conviction that a *specific* conspiracy theory is true and—logically—the “officially” presented explanation intentionally wrong. In this article, the “secret plot” the citizens may or may not believe in concerns the *Corona pandemic*. As we specifically investigate the belief that the pandemic is a hoax, essentially this paper is about Corona deniers.

Conspiracy theories are not a new thing in German political discourse. Indeed, a significant minority has held beliefs that questioned the motifs of government action and suspected undisclosed forces behind actions in several contexts before (Anton et al., 2014; Freitag, 2014; Krüger and Seiffert-Brockmann, 2017). Roose (2020) finds that roughly ten percent of the German population subscribe to conspiracy theories of various kinds. This is important as some studies suggest that COVID-19 conspiracy beliefs may be linked to a general conspiracy thinking (Gemenis, 2021). In this vein, the corona pandemic may have exacerbated existing general conspiracy orientations (Schließler et al., 2020). Those who are suspicious of the government in general could thus be expected to react particularly negatively if their freedoms were curtailed to such a great extent as the pandemic required. A subscription to views perpetuating doubt about the origins of COVID-19 may therefore come easy to someone who is already leaning toward majorly distrusting government actions.

Arguably, that mechanism is enhanced when people with very closed and specific views exchange those largely with others who confirm them. Not just since lockdown measures have been implemented, but obviously increasingly since, much communication has taken place *via* electronic channels. Social media in particular was the main pathway for COVID-19 conspiracy theory claims to be distributed to a wide audience (Schüler et al., 2021). But much of the emergent exchanges *via* social media channels then occurred within isolated bubbles in which facts were typically ignored (Scharkow et al., 2020) and consequently suspicious views found a strong confirmation by others also holding them. Such isolated bubbles were thus likely to enhance the shared construction of conspiracy narratives (Goreis and Kothgassner, 2020; Rocha Dietz et al., 2021).

There is a growing body of research on which people adopt conspiracy theories and why (for a review, see Douglas et al., 2019). For the issue of the COVID-19 pandemic specifically, previous research has identified a number of individual characteristics that are associated with conspiracy thinking (van Mulukom et al., 2022). A first set of characteristics is *socio-demographic* in nature. In line with the idea of cognitive mobilization, in Germany (Schließler et al., 2020) and in Poland (Duplaga, 2020) support for pandemic-related conspiracy beliefs is more widespread among the low educated. The study by Schließler et al. (2020) also emphasizes low income as a significant determinant, which might indicate that a low social status generally makes people susceptible to corona conspiracy beliefs. For age, there is evidence that support for conspiracy thinking is stronger in younger age groups (Duplaga, 2020). Other studies point to a gender gap, with men being more likely to endorse COVID-19 conspiracy theories (Cassese et al., 2020). However, findings from these studies are not easy to compare due to differences in methodology and variables included.

Research on participants in German anti-Corona demonstrations (a significant number of whom, albeit not all, share conspiracy views) suggests that next to socio-demographics, various political attitudes have also to be taken into account (Frei and Nachtwey, 2021; Koos, 2021). An important debate is about which *political camps* are breeding grounds for COVID conspiracy beliefs. While Schließler et al. (2020), for example, found a greater propensity to hold pandemic conspiracy views both for the far right and left, other scholars singled out the far right (Nachtwey et al., 2020; Spöri and Eichhorn, 2021), in line with findings from international research (Prichard and Christman, 2020; Frindte, 2021).

Another attitudinal dimension found to be influential in several countries is *(dis-)trust in institutions* (Đorđević et al., 2021; Stecula and Pickup, 2021). Conspiracy believers often have a very low level of trust in the government (for Germany see Hövermann, 2020), and in state institutions more widely. The distrust can stretch beyond the state and connect to a broader populist anti-elite sentiment, as Stecula and Pickup (2021) demonstrate for the USA, or to authoritative experts such as scientists (Eberl et al., 2021). There is also mounting evidence on the role of consuming a very narrow set of media (in the USA, mainly conservative media outlets), especially social media channels (Goreis and Kothgassner, 2020; Allington et al., 2021). There is one more factor: distrust in public broadcasters is not a new phenomenon in Germany (Hagen, 2015; Krüger and Seiffert-Brockmann, 2017), yet such distrust can further exacerbate the propensity for conspiracy beliefs.

Human values and anti-social orientations might also factor in (Enders et al., 2021). Conspiracy theories on the pandemic find more support amongst people who feel threatened and perceive a loss of control (Kim and Kim, 2020). Arguably, this can fuel a particularism that puts one's own personal interests and those of the like-minded ingroup above the common good. One can see that in the value profiles for COVID-19 conspiracy theory supporters and non-supporters. While the former score low on conformity, the latter score high on universalism (Spöri and Eichhorn, 2021) and collectivism (Biddlestone et al., 2020). This suggests that pandemic conspiracy believers reject value orientations that impinge on self-centered values.

What are the research gaps? For one, more research is needed in order to accumulate knowledge on the correlates of (pandemic) conspiracy beliefs. In this context, studies which include a wide range of socio-demographic, political, and attitudinal characteristics are particularly helpful. Next and most importantly, the lion's share of previous research is cross-sectional. Little is known, therefore, how stable—or malleable—COVID-19 conspiracy beliefs have been over the course of events. This is particularly important in the context of the Coronavirus pandemic, since the rising numbers of infected and dead in Germany and elsewhere in the world made it increasingly difficult to deny the obvious: that there *is* an ongoing pandemic.

Against this backdrop, the study aims to contribute to the research field in two ways. The first goal is to thoroughly examine who the conspiracy believers in Germany are, both in terms of socio-demography and political ideology (what we summarize as the socio-political profile) and in terms of attitudinal dispositions (the attitudinal profile). The second goal is to shed light on the individual-level changes in conspiracy beliefs that happened from the first (2020) to the second (2021) year of the pandemic. The panel data that we are going to use—described in detail in the next section—allow to explore such dynamics, and to identify the group of consistent COVID conspiracy believers that stick to their denial of the pandemic over time. Our main contribution, therefore, is to provide insights on which characteristics distinguish the *core group* of conspiracy believers in Germany.

## Data, variables, and method

### Data

The present paper draws on the German samples from the first two waves of a panel study fielded in Germany and the United Kingdom. The panel study was designed and conducted for the purposes of the “Values in Crisis” project, a joint research endeavor of the Otto von Guericke University Magdeburg (Germany), the University of Edinburgh (Scotland, UK) and Jacobs University Bremen (Germany), in cooperation with the think tank d|part (Germany), funded by the Volkswagen Stiftung. Taking the Corona pandemic as a natural experiment, the project attempts to investigate value change in times of major crises. The first wave was fielded at the beginning of the pandemic (April 24–May 19, 2020), the second wave—approximately 10 months later (February 15–March 15, 2021). The data were collected in both countries by Bilendi GmbH, a market and opinion research company specializing on online data collection among a large pool of panelists. The panel study employs quota sampling with regard to the composition of the respective national population of age 16 and above along biological sex, age, educational attainment (highest level achieved), and region (federal state in the case of Germany). The panel study further applied cross-quotas for age within a region and educational attainment within a region in order to ensure sufficient representation of the target populations within sub-strata, too. Small batches of participants were invited at regular intervals in order to ensure that the target sample characteristics would be met best: Upon detecting that certain groups were underrepresented at a certain stage, invites to these groups were increased. The samples obtained meet the target characteristics to an extent that the application of sample weights does not substantially change the results. To exemplify, the computed weights shift the frequency distributions of key socio-demographic variables by less than one percentage point. Indeed, as research has shown (Baker et al., 2010; Rada and Martín, 2014), quota samples based on large, high-quality panels allowing for detailed stratification beyond basic demographics perform very well.

Concerning the German data, the 2009 participants who took part in the first wave of data collection were invited to participate in the second wave, too. Key socio-demographic characteristics were re-collected in order to ensure that the same persons participated in both waves. Respondents for whom these characteristics could not be matched across both waves, were not included in the panel sample. The latter consists of 1,280 respondents. This results in a validated retention rate of just over 60%. Minor biases in the pattern of attrition were accounted for by longitudinal weights, adjusting thereby the panel sample to the target population parameters. The panel sample serves as the working sample for the analyses to be presented. Due to the questionnaire design (forced choice), the data were not affected by missing values.

### Variables

#### Corona conspiracy beliefs

Respondents' belief in Corona conspiracy theories was measured with the item: “*The social media are full of stories saying that the Corona pandemic is a hoax and that all the lockdown measures are a hysteric overreaction. Do you believe in these stories?*”. The question is formulated in an intentionally pointed way to ensure that respondents genuinely subscribe to an extreme position associated with the denial of the pandemic rather than merely expressing doubts about it. As such, the item is a reflection of the public debate on the issue, particularly at the onset of the pandemic. Its aim was to identify respondents who subscribed to the two dominant and related conspiracy narratives at the time: questioning the existence or nature of the virus in the first place, and, in consequence, opposing anti-COVID measures. Intentionally double-barrelled, the item sets a high bar for agreement with the statement, excluding those who only disagree with the scope of government measures (but do not reject the existence of the pandemic *per se*) or those who may generally agree with the hoax narrative without a negative view on the measures (the latter case is presumably way less frequent than the former).

Based on the responses to the question from the second wave of data collection, we consider respondents who answered “Yes” as Corona deniers and those who answered “No” as Corona realists. Beside a static account on the spread of Corona denial, we examine its change from Wave 1 to Wave 2. The joint pattern of responses across both waves produces a four-fold typology: consistent realists (“No” in both waves), former deniers (“Yes” in Wave 1, “No” in Wave 2), new deniers (“No” in Wave 1, “Yes” in Wave 2), and consistent deniers (“Yes” in both waves).

#### Socio-political characteristics

In order to account for respondents' socio-political profile, the analyses consider the following characteristics (categories in brackets, reference category in italics): biological sex (male, *female*); age group (16–34 years, *35–64 years*, 65 years and above); having a partner (yes—married or living together as married, *no*—divorced, separated, widowed, or single); having children (yes, *no*); education (lower, *intermediate*, high); income class^1^ (low, lower-middle, *middle*, upper-middle, high); type of settlement (village, *town*, city or suburb); region of Germany (East, *West*); political views^2^ (left-wing, *center*, right-wing); and whether the respondent has been affected by COVID-19^3^ (yes, *no*).

#### Attitudinal controls

In addition to the socio-political characteristics, the analyses account for a number of attitudes and dispositions that can be plausibly assumed to relate to Corona conspiracy beliefs. First, we consider the extent of distrust in institutions. Respondents were asked to rate their confidence in the country's government, health sector, institutions as a whole, scientific experts, and public service broadcasters. Each item had a four-point answering scale, ranging from 1 (a great deal) to 4 (none at all). Thus, a higher numeric code stands for greater distrust. The five items form a unifactorial solution and have sufficiently high loadings between 0.79 (health sector) and 0.85 (institutions as a whole). Cronbach's alpha coefficient for internal consistency is at α = 0.87. We, therefore, subsumed the items into an index of institutional distrust by taking their arithmetic mean.

Second, we look at whether a perception of social media as more credible than traditional media is associated with Corona conspiracy beliefs. The exact item wording reads: “How credible do you think are the social media, like Twitter and Facebook, compared to the traditional media, like TV and newspapers?”. The original answering scale was reversed to range from 1 (traditional media are most credible) over 3 (both the same) to 5 (social media are most credible).

Third, we check whether value orientations previously shown to be associated with COVID-19 conspiracy beliefs, namely universalism and conformity (Spöri and Eichhorn, 2021), are indeed relevant in identifying Corona deniers. Both values stem from Schwartz' theory of basic human values (Schwartz, 1992). Conformity pertains to a preference to avoid actions, inclinations, and impulses that can harm others or violate social expectations and norms. Universalism pertains to a preference for tolerance and understanding as well as the protection of people's welfare and nature. Each value type was measured with the respective items from the Schwartz value inventory in the European Social Survey. Following the established methodology, respondents' ratings on the items were first ipsatized before computing the scores on the two value types. The resulting scores have been truncated to a four-point scale, with a higher number standing for a stronger preference for the respective value.

Finally, we consider specific attitudes and dispositions related to the topic. On the one hand, we account for respondents' emphasis on freedom as compared to health. The exact item wording reads: “There is much debate about what should take top priority in times of the pandemic: the freedom of citizens, or the protection of health? In your view, what should take top priority?”. The original response scale was reversed to range from 1 (health) over 3 (both equally important) to 5 (freedom). On the other hand, we account for respondents' affinity for, what we call, myths about Corona. Respondents were asked to state to what extent they agree or disagree with the following items: “The virus is manmade.”, “The spread of the virus is a deliberate attempt by one nation to destabilize others.”, and “The spread of the virus is a deliberate attempt by a group of powerful people to make money.” Each item was accompanied with a Likert scale from 1 (strongly disagree) to 5 (strongly agree). The items form a unifactorial solution and have loadings from 0.86 to 0.93. Cronbach's alpha coefficient of internal consistency was found at α = 0.87. We, therefore, subsumed the three items into an index of affinity for Corona myths by taking their arithmetic mean.

Table A1 provides descriptive statistics for all variables used in the analyses.

### Method

Starting with a brief descriptive account on the spread of Corona denial, as measured in Wave 2, and on the change in Corona conspiracy beliefs from Wave 1 to Wave 2, the paper proceeds to a series of binary logistic regressions aiming to uncover the socio-political profile of Corona deniers, as compared to Corona realists, accounting for their attitudes and dispositions in an additional step. Applying a multinomial logistic regression on the four-fold typology of change in Corona conspiracy beliefs, the paper also offers a fine-grained look into this profile. The use of the logit link in the logistic regressions makes it possible, *via* exponentiation, to present the regression coefficients from the linear prediction of the log-odds in the form of odds ratios (binary scenario) or relative risk ratios (multinomial scenario), respectively. The latter two estimates can be interpreted as multiplicative factors to the odds of being a (specific type of) denier relative to the realists.

In addition, we compare the attitudinal profile of the Corona deniers using independent-samples *t*-tests and one-way analyses of variance followed by the conservative Scheffé *post-hoc* test. All analyses were performed in Stata 17 (StataCorp, 2021).

## Results

### Changes in Corona conspiracy beliefs, 2020–2021

**Figure 1:**
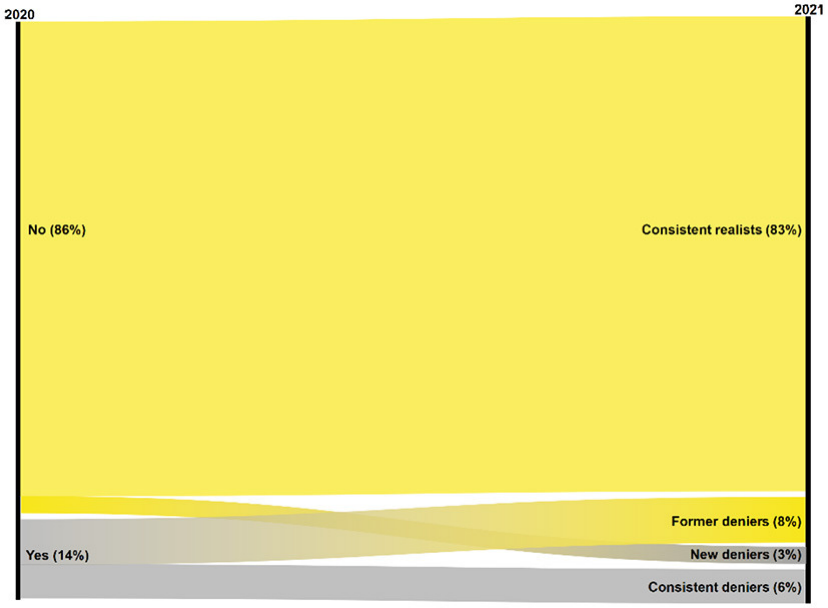
Dynamics of Corona denial: changes from 2020 to 2021. The figure presents the relative frequencies (%) of yes-no responses to the hoax item in 2020 and how these changed in 2021. See section “Variables” for the exact item wording item and an elaboration of the four-fold typology in 2021.

Most Germans do *not* believe that Corona pandemic is a hoax. Yet, a non-negligible minority does so, albeit at a declining rate. At the onset of the pandemic around April–May 2020, 86% of the respondents aged 16 and older disagreed with the statement that the pandemic is a hoax and the government response a hysteric overreaction, whereas 14% agreed. Ten months later around February–March 2021, after two lockdowns and cumulated deaths in the order of 70,500 (March 1, 2021), the group of Corona deniers has shrunk to nine percent. This aggregate comparison, however, does not showcase the full extent of the dynamic observable at the individual level (see Figure 1). Considering the pattern of responses to the hoax item across both waves of our panel survey, we identify four groups of citizens. Eighty-three percent disagreed that the Corona pandemic is a hoax both in 2020 and in 2021, thereby constituting the large group of *consistent realists*. The remaining 17% of the respondents agreed with the hoax item in at least one of the two waves, thus forming three groups of deniers. Eight percent can be considered *former deniers* as they agreed with the hoax item in 2020, but were not any longer of this opinion by 2021. The other three percent of the respondents form the group of *new deniers*: having initially considered the Corona pandemic to be real, they denied it in 2021. Finally, those who agreed with the hoax item in both years represent six percent of all respondents and constitute the group of *consistent deniers*.

Table 1 provides an overview of the representation of the four groups in the former regions of West and East Germany. In the Western part of the country, the distribution across the four groups is almost identical to that of Germany as-a-whole (see Table 1). This is probably due to the fact that the Western population constitutes the larger share of the population with about 67 million citizens (vs. about 13 million in former East Germany), and thus dominates the all-German distribution. In the Eastern part, too, a great majority consistently accepted Corona as a fact; yet, this majority is smaller than in the Western part (75 vs. 84%, respectively). Accordingly, the three groups of Corona deniers are a bit larger in the East than in the West, especially the consistent deniers (11 vs. 5%, respectively). Given these regional differences, we supplement the main analysis with a regional analysis specifically for the East.

**Table 1:** Dynamics in Corona conspiracy beliefs in East and West Germany.

	**All Germany**		**West Germany**		**East Germany**	
	***N***	**%**	***N***	**%**	***N***	**%**
Consistent realists	1,064	83.1	897	84.8	167	75.2
Former deniers	95	7.4	75	7.1	20	9.0
New deniers	44	3.4	34	3.2	10	4.5
Consistent deniers	77	6.0	52	4.9	25	11.3

### The socio-political profile of Corona deniers

We now proceed to a binary logistic regression of Corona denial in order to identify the basic *socio-political profile* of those thinking of the pandemic as a hoax in at least one of the two survey waves (see Table 2, Model 1). This base model accounts for about ten percent of the individual differences in the probability to deny the Corona pandemic, with a number of characteristics yielding significant effects. According to the sizes of the odds ratios, respondents' income class is the most influential characteristic. The odds to deny the pandemic are almost three times higher among respondents living on low income as compared to those with middle income (*OR* = 2.718, *p* ≤ 0.01). Country region shapes conspiracy beliefs just as strongly as income does: Residents of former East Germany have nearly three times higher odds to deny the pandemic than those of West Germany (*OR* = 2.708, *p* ≤ 0.01). Age emerges as the third most important characteristic: Compared to respondents of age 35–64 years, the youngest group has roughly double the odds to consider the pandemic a hoax (*OR* = 1.988, *p* ≤ 0.01). Whereas, the odds of Corona denial tend to be roughly 30% smaller among the elderly (*OR* = 0.717), the latter group does not differ significantly from respondents of middle age. A fourth important characteristic is the respondents' political identification: in comparison to centrists. The odds of Corona denial are about 64% lower among left-wingers (*OR* = 0.365, *p* ≤ 0.01) and almost twice as high among right-wingers (*OR* = 1.965, *p* ≤ 0.01). The last influential characteristic is education: respondents with low education have about 1.7 times greater odds to deny the pandemic than their fellow citizens with medium-level education (*OR* = 1.676, *p* ≤ 0.05). The results point to virtually no difference between respondents with medium-level education and those with higher education. None of the other characteristics considered in the base model—biological sex, having a partner, having children, size of settlement, or having been affected by COVID-19—are significantly related to (dis-)agreement with the hoax item. In a nutshell, the socio-economic profile of the “typical” Corona denier in Germany is characterized by low income, residence in former East Germany, young age (below 35), self-identification as right-winger, and low education.

**Table 2:** Binary logistic regression of Corona denial on socio-political characteristics.

	**Model 1**					
	**All Germany**		**West Germany**		**East Germany**	
**Sex**: male	1.145		1.099		1.282	
	(0.66)		(0.40)		(0.58)	
**Age group**						
16–34 years	1.988	^***^	2.092	^**^	2.408	
	(2.59)		(2.49)		(1.30)	
65+ years	0.717		0.737		0.757	
	(−1.37)		(−1.03)		(−0.58)	
**Partner:** yes	1.014		1.528		0.322	^**^
	(0.06)		(1.56)		(−2.53)	
**Children:** yes	1.066		0.959		1.297	
	(0.28)		(−0.15)		(0.51)	
**Education**						
Lower	1.676	^**^	1.811	^**^	0.909	
	(2.03)		(2.02)		(−0.17)	
High	1.157		1.369		0.571	
	(0.56)		(1.05)		(−0.87)	
**Income class**						
Low	2.718	^***^	2.167	^*^	12.490	^**^
	(2.69)		(1.90)		(2.24)	
Lower-middle	1.747		1.375		5.715	
	(1.53)		(0.80)		(1.57)	
Upper-middle	0.889		0.620		4.596	
	(−0.28)		(−1.04)		(1.31)	
High	1.803		1.490		3.056	
	(1.32)		(0.83)		(0.82)	
**Settlement**						
City or suburb	0.819		0.770		1.296	
	(−0.85)		(−0.93)		(0.54)	
Village	1.034		1.196		0.759	
	(0.13)		(0.60)		(−0.46)	
**Germany:** East	2.708	^***^				
	(4.24)					
**Political views**						
Left-wing	0.365	^***^	0.432	^**^	0.217	^***^
	(−3.58)		(−2.53)		(−2.67)	
Right-wing	1.965	^***^	1.879	^**^	2.463	
	(2.88)		(2.37)		(1.62)	
COVID-19 affected	1.067		1.127		0.637	
	(0.29)		(0.46)		(−0.90)	
Intercept	0.044	^***^	0.040	^***^	0.065	^**^
	(−7.22)		(−6.46)		(−2.29)	
Pseudo-*R^2^*	0.103		0.092		0.191	

The table shows the results from a binary logistic regression of Corona denial on socio-political characteristics for Germany as a whole (*N*_*Total*_ = 1,280), and separately for West Germany (*N*_*West*_ = 1,058) and East Germany (*N*_*East*_ = 222). The presented coefficients are odds-ratios with *z*-scores in brackets. Significance of the estimates in two-sided tests:

* *p* ≤ 0.10,

** *p* ≤ 0.05,

*** *p* ≤ 0.01. Reference categories: Sex: female, Age group: 35–64 years, Partner: no, Children: no, Education: intermediate, Income class: middle, Settlement: town, Germany: West, Political views: center.

In a second step, we differentiate the analysis along the four types of Corona conspiracy believers that arise in a longitudinal perspective: Are there differences among former, new, and consistent Corona deniers, as compared to the large group of consistent realists who have accepted the pandemic as a reality from the very beginning? Table 3 shows the results from a multinomial logistic regression in the form of the so-called relative risk ratios (*RRR*). Just as in the binomial logistic regression, (young) age, (low) education, (lower) income, residing in East Germany, and having a right-wing political orientation turn out to be risk factors for Corona denial in any form; yet, with a clear gradient across the three denier groups. Regarding age, the young are at a 1.7 times higher risk to be former deniers (*RRR* = 1.713, *p* ≤ 0.10) and at a 3.6 times higher risk to be new deniers (*RRR* = 3.581, *p* ≤ 0.10) than respondents of middle age. The elderly, in contrast, tend to be at a consistently lower risk of denying the pandemic in any form, but the protective effect of advanced age is only significant—and only marginally so—against being a former denier (*RRR* = 0.407, *p* ≤ 0.10). The low educated respondents are at 1.7 times greater risk to be consistent deniers (*RRR* = 1.660, *p* ≤ 0.10) than respondents with intermediate education. High education emerges as a marginally significant protective factor against being a former denier (*RRR* = 0.576, *p* ≤ 0.10). Living on low or lower-middle income is associated with a greater risk to be a consistent denier: The relative risk to end up in this group is four times higher for respondents living on low income (*RRR* = 4.126, *p* ≤ 0.01) and 2.4 times higher for respondents living on lower-middle income (*RRR* = 2.438, *p* ≤ 0.10). In comparison to residents of West Germany, East Germans are consistently at a greater risk of Corona denial in any form: They have a 1.7 times greater risk to be former deniers (*RRR* = 1.675, *p* ≤ 0.10), 2.3 times greater risk to be new deniers (*RRR* = 2.261, *p* ≤ 0.05), and 3.2 times greater risk to be consistent deniers (*RRR* = 3.190, *p* ≤ 0.01). As to political views, a left-wing identification acts as a protective factor against any form of Corona denial as compared to a centrist orientation: Left-wingers are at a 35% lower risk to be former deniers (*RRR* = 0.652, *p* ≤ 0.10), about 60% lower risk to be new deniers (*RRR* = 0.391, *p* ≤ 0.05), and 68% lower risk to be consistent deniers (*RRR* = 0.322, *p* ≤ 0.01). Respondents of a right-wing political orientation are, in contrast, at 2.3 times greater risk to be consistent deniers (*RRR* = 2.343, *p* ≤ 0.01) than centrists. In a nutshell: The composition of the group of former deniers is characterized with an overrepresentation of young East Germans and an underrepresentation of the highly educated respondents and left-wingers; that of new deniers—with a stronger representation of young East Germans and a stronger underrepresentation of left-wingers; that of consistent deniers—with the strongest representation of East Germans, on top of that respondents with low education, low to lower-middle income, and right-wing political orientation as well as the strongest underrepresentation of left-wingers.

**Table 3:** Multinomial logistic regression of type of deniers on socio-political characteristics.

	**Model 2**					
	**Former**		**New**		**Consistent**	
**Sex:** male	1.238		1.218		1.179	
	(0.96)		(0.61)		(0.66)	
**Age group**						
16–34 years	1.713	^*^	3.581	^***^	1.343	
	(1.89)		(3.33)		(0.82)	
65+ years	0.407	^***^	0.515		0.704	
	(−3.24)		(−1.48)		(−1.23)	
**Partner:** yes	1.125		1.543		0.833	
	(0.47)		(1.18)		(−0.68)	
**Children:** yes	0.942		0.826		1.235	
	(−0.24)		(−0.52)		(0.73)	
**Education**						
Low	1.476		1.944		1.660	^*^
	(1.39)		(1.59)		(1.65)	
High	0.576	^*^	0.955		1.216	
	(−1.76)		(−0.11)		(0.60)	
**Income class**						
Low	1.716		1.812		4.126	^***^
	(1.37)		(1.12)		(2.76)	
Lower-middle	1.506		1.273		2.438	^*^
	(1.10)		(0.47)		(1.75)	
Upper-middle	1.662		0.617		1.297	
	(1.34)		(−0.80)		(0.46)	
High	0.456		1.337		2.155	
	(−1.17)		(0.47)		(1.25)	
**Settlement**						
City or suburb	0.945		0.783		0.834	
	(−0.22)		(−0.65)		(−0.63)	
Village	1.234		1.041		1.083	
	(0.74)		(0.10)		(0.25)	
**Germany:** East	1.675	^*^	2.261	^**^	3.190	^***^
	(1.85)		(2.07)		(4.13)	
**Political views**						
Left-wing	0.652	^*^	0.391	^**^	0.322	^***^
	(−1.65)		(−2.21)		(−3.09)	
Right-wing	1.210		1.473		2.343	^***^
	(0.64)		(1.01)		(3.02)	
COVID-19 affected	0.771		1.543		0.780	
	(−1.03)		(1.28)		(−0.85)	
Intercept	0.071	^***^	0.019	^***^	0.024	^***^
	(−5.88)		(−6.16)		(−6.37)	
Pseudo-*R^2^*	0.089					

The table shows the results from a multinomial logistic regression of type of Corona deniers on socio-political characteristics (*N* = 1,280). The presented coefficients are relative risk ratios (in comparison to the consistent realists) with *z*-scores in brackets. Significance of the estimates in two-sided tests:

* *p* ≤ 0.10,

** *p* ≤ 0.05,

*** *p* ≤ 0.01. Reference categories: Sex::female, Age group::35–64 years, Partner::no, Children::no, Education::intermediate, Income class::middle, Settlement::town, Germany::West, Political views::center.

### The attitudinal profile of Corona deniers

Next, we add a range of *attitudinal* characteristics to the base model, each specified in a separate model, in order to uncover attitudes and dispositions feeding into the Corona conspiracy beliefs (Table 4, Models 3–7). With the exception of Model 5 (the human values model), the pseudo-*R*^2^ measure is more than twice as high as in the base model, which indicates that Corona denial indeed forms a tightly knit syndrome with other attitudes. Nevertheless, the socio-political variables identified as relevant in the base model are surprisingly robust, when attitudes are considered one at a time: The effects of young age, living in East Germany, and political ideology (both far right and left) are significant in all models (5/5); that of low income in all but one model (4/5); and that of low education in all but two models (3/5). When the entire set of attitudes is added to the base model in one go (results not shown), the socio-political characteristics—bar age and living in East Germany—lose power.

**Table 4:** Binary logistic regression of Corona denial on socio-political and attitudinal characteristics.

	**Model 3**		**Model 4**		**Model 5**		**Model 6**		**Model 7**	
**Sex:** male	**1.221**		**1.173**		**1.104**		**0.900**		**1.108**	
	(0.93)		(0.73)		(0.48)		(−0.47)		(0.46)	
**Age group**										
16–34 years	2.167	^***^	1.820	^**^	1.853	^**^	2.076	^**^	1.903	^**^
	(2.74)		(2.13)		(2.29)		(2.54)		(2.21)	
65+ years	0.870		0.774		0.736		0.936		0.963	
	(−0.54)		(−0.97)		(−1.24)		(−0.25)		(−0.14)	
**Partner:** yes	0.963		0.958		1.058		1.129		0.853	
	(−0.16)		(−0.18)		(0.25)		(0.50)		(−0.66)	
**Children:** yes	1.173		1.025		1.039		1.074		0.824	
	(0.65)		(0.10)		(0.16)		(0.29)		(−0.77)	
**Education**										
Low	1.459		1.592	^*^	1.754	^**^	1.935	^**^	1.116	
	(1.39)		(1.71)		(2.18)		(2.40)		(0.39)	
High	1.284		1.278		1.130		1.221		1.422	
	(0.89)		(0.87)		(0.46)		(0.70)		(1.22)	
**Income class**										
Low	1.976	^*^	1.963	^*^	2.551	^**^	2.562	^**^	1.754	
	(1.73)		(1.71)		(2.51)		(2.35)		(1.40)	
Lower-middle	1.551		1.503		1.682		1.726		1.539	
	(1.13)		(1.06)		(1.41)		(1.39)		(1.10)	
Upper-middle	0.867		0.843		0.827		0.891		0.924	
	(−0.33)		(−0.39)		(−0.46)		(−0.26)		(−0.18)	
High	2.167	^*^	1.921		1.648		2.184		1.702	
	(1.66)		(1.39)		(1.11)		(1.64)		(1.10)	
**Settlement**										
City or suburb	0.787		0.751		0.792		0.720		0.818	
	(−0.95)		(−1.13)		(−0.99)		(−1.30)		(−0.78)	
Village	1.066		0.975		1.024		0.853		0.934	
	(0.23)		(−0.09)		(0.09)		(−0.56)		(−0.24)	
**Germany:** East	2.231	^***^	2.281	^***^	2.611	^***^	2.635	^***^	2.372	^***^
	(3.16)		(3.22)		(4.01)		(3.78)		(3.29)	
**Political views**										
Left-wing	0.491	^**^	0.447	^***^	0.388	^***^	0.477	^**^	0.590	^*^
	(−2.43)		(−2.70)		(−3.30)		(−2.48)		(−1.74)	
Right-wing	1.624	^*^	2.076	^***^	1.834	^**^	1.672	^**^	1.705	^**^
	(1.92)		(2.86)		(2.54)		(2.00)		(2.04)	
COVID-19 affected	1.343		1.234		1.114		1.186		1.120	
	(1.24)		(0.87)		(0.47)		(0.70)		(0.46)	
Institutional distrust	4.533	^***^								
	(9.07)									
Social/trad. media			2.886	^***^						
			(9.51)							
Conformity					0.718	^***^				
					(−3.47)					
Universalism					0.857					
					(−1.53)					
Freedom/health							2.602	^***^		
							(9.61)			
Corona myths									2.829	^***^
									(10.26)	
Intercept	0.001	^***^	0.003	^***^	0.144	^***^	0.003	^***^	0.003	^***^
	(−10.55)		(−10.15)		(−3.68)		(−10.21)		(−10.31)	
Pseudo-*R^2^*	0.218		0.237		0.125		0.243		0.268	

The table shows the results from binary logistic regressions of Corona denial on socio-political characteristics and various attitudes (*N* = 1,280). The presented coefficients are odds-ratios with *z*-scores in brackets. Significance of the estimates in two-sided tests:

* *p* ≤ 0.10,

** *p* ≤ 0.05,

*** *p* ≤ 0.01. Reference categories: Sex: female, Age group: 35–64 years, Partner: no, Children: no, Education: intermediate, Income class: middle, Settlement: town, Germany: West, Political views: center.

Institutional distrust is associated with higher odds to deny the pandemic: A one-point increase in distrust in institutions raises four to five times the odds of denial (*OR* = 4.533, *p* ≤ 0.01). Each of the items that flowed into the institutional distrust index has a comparable effect, when used separately (results not shown). Trusting social media more than traditional media has a similar, though slightly weaker effect (Model 4). A one-point stronger preference for social media over traditional media increases the odds of Corona denial almost three times (*OR* = 2.886, *p* ≤ 0.01). The basic human values of conformity and universalism, in contrast, play only a minor role (Model 5). While universalism seems to be statistically unrelated, people who endorse conformity more strongly are significantly less likely to consider the pandemic a hoax; a one-point stronger preference for conformity reduces the odds of Corona denial by almost 30 % (*OR* = 0.718, *p* ≤ 0.01). In other words, Corona deniers can be characterized as “non-conformists”. Moving on to pandemic-specific attitudes, the preference for individual freedom over health concerns (Model 6) is strongly associated with Corona denial: A one-point stronger preference for freedom raises the odds of denial 2.6 times (*OR* = 2.602, *p* ≤ 0.01). Admittedly, though, it is difficult to say here what is cause and what is effect. Finally, and expectedly, an inclination to believe in specific Corona myths contributes to supporting the hoax and overreaction argument (Model 7). The odds of denial are 2.8 times higher at each one-point increase in the belief in Corona myths (*OR* = 2.829, *p* ≤ 0.01). In fact, a comparison of the pseudo-*R*^2^ of each extended model with that of the base model reveals that the most influential attitudinal characteristics in determining the probability of Corona denial are (in this order): belief in Corona myths, preference for freedom over health, preference for social media over traditional media, and institutional distrust. When the entire set of attitudes is added to the base model in one go (results not shown), all attitudinal variables remain significant, except institutional distrust, which is cannibalized by the more powerful trust in social media variable.

Now, does the mindset of the three *groups* of deniers—former, new, and consistent—differ? A series of one-way ANOVA analyses provides the answer (see summary of findings in Table 5; full results in Table A2). There are statistically significant differences in all dispositions examined between at least two of the three groups, except for universalism. As a rule of thumb, consistent deniers have the most extreme mindset. In comparison to the other two groups, they distrust institutions most strongly; have the highest level of trust in social media (here, the new deniers are on par); endorse conformity the least (on par with former deniers); prefer freedom over health most strongly; and endorse specific Corona myths the most (here, the new denies are on par). Hence, there is quite a gradient of “extreme” thinking running from consistent over new to former Corona deniers.

**Table 5:** Differences in attitudes across groups of Corona deniers.

**Attitude/Disposition**	**Lowest**				**Highest**
Institutional distrust	New	=	Former	<	Consistent
Social over traditional media	Former	<	New	=	Consistent
Conformity	Consistent	=	Former	<	New
Universalism	Consistent	=	New	=	Former
Freedom over health	Former	=	New	<	Consistent
Corona myths	Former	<	New	=	Consistent

The table summarizes the results from a series of one-way analyses of variance, each performed for a particular attitude/disposition by type of Corona deniers (see Table A2 for full results). An < or > sign indicates a statistically significant difference in the respective direction. An = sign indicates no significant difference between the respective two groups.

### A final look: East German peculiarities?

Since the data indicated a larger reservoir of Corona deniers in the Eastern part of the country, we re-estimated selected models for West and East Germany separately. As the results for West Germany and Germany as-a-whole are very similar (for the reason given above), we primarily focus on East Germany. We first revisit the socio-political profile of hoax believers (see Table 2, Model 1-East and Model 1-West). A first peculiarity concerns political ideology: Unlike in the West, right-wing identification is not a significant determinant in the East. This suggests that Corona denial is more widespread in the East even in the centrist political camp (which serves as the reference group in the regression), whereas it is confined to small pockets of right-wingers in the West. Left-wingers are significantly underrepresented among Corona deniers, even more so in the East (*OR* = 0.217) than in the West (*OR* = 0.432), probably a matter of distinction in an opinion climate in which conspiracy beliefs are more acceptable. Second, there are no age differences in the East. Instead, partner status plays a role, with those who have a partner having almost 70% lower odds in considering Corona a hoax (*OR* = 0.322, *p* ≤ 0.05). Finally, denying the pandemic is clearly a low-income matter: Low-income earners have twelve times greater odds to support the hoax item (*OR* = 12.490, *p* ≤ 0.01) than medium income earners. Financial dissatisfaction or feelings of relative deprivation, therefore, could motivate Corona deniers in the East.

With our final analysis, we examine whether the three East German groups of Corona deniers each differ from their West German counterparts in terms of their attitudinal profile with respect to institutional distrust, trust in social media, human values (conformity and universalism), preference for freedom, and specific Corona myths. The short answer is: “no” (see summary of results in Table 6). Neither in the group of consistent deniers nor in the group of former deniers is there any statistically significant difference between East and West Germany. For the group of new deniers, there is one single difference: East Germans endorse universalism more than West Germans [*t*_(42)_ = 2.29, *p* = 0.027]. For all other attitudes, this group is similar in the East-West comparison. This leaves us with the following conclusions on the East-West-issue. First, Corona conspiracy beliefs are more widespread among East Germans. Second, while the socio-political profile of Corona deniers shows some peculiarities, mindsets do not: East German deniers are not attitudinally “more extreme” than their West German counterparts.

**Table 6:** Attitudinal profiles of Corona deniers across East and West Germany.

**Attitude/Disposition**	**Former**	**New**	**Consistent**
Institutional distrust	East = West	East = West	East = West
Social over traditional media	East = West	East = West	East = West
Conformity	East = West	East = West	East = West
Universalism	East = West	East > West	East = West
Freedom over health	East = West	East = West	East = West
Corona myths	East = West	East = West	East = West

The table summarizes the results from a series of independent-samples *t*-tests of the respective attitude/disposition, comparing respondents from West and East Germany, within a specific type of Corona deniers (full results are available upon request). A < or > sign indicates a statistically significant difference at *p* ≤ 0.05 in a two-sided test. An = sign indicates no statistically significant difference.

## Discussion

Like others before, the study at hand sought to shed light on both the extent and the socio-political and attitudinal profile of citizens who consider the Coronavirus pandemic a hoax, examining the case of Germany. Yet unlike most studies, we used two waves of panel data collected in spring 2020 and spring 2021, which allowed us to examine individual-level changes and thus to identify different types of Corona conspiracy supporters: former, new, and consistent. Considering the dynamics of COVID-19 denial and differentiating between groups is important, as their socio-political and attitudinal profiles are not uniform. We consider the following results to be most important.

First, as the pandemic unfolded, the camp of conspiracy believers—a clear minority of the German population—became smaller, as it lost more followers than it gained new ones. This development was the expected pattern for a conspiracy belief that denies an—unfortunately—powerfully unfolding medical reality, with skyrocketing numbers of COVID-19 infections and an increasing death toll. Still, a small minority of six percent considered the pandemic a hoax in 2020 *and* still in 2021, and, even more irrationally, three percent converted to that idea in 2021.

Second, our study confirms that socio-demographic characteristics such as age, education, and income as well as political ideology are associated with the propensity to believe in COVID-19 conspiracy theories (cf. the review by van Mulukom et al., 2022). In comparison to previous studies, an especially notable result concerns the role of political self-placement. While one available study had suggested that in Germany conspiracy beliefs about the pandemic are to be found at *both* edges of the political spectrum (Schließler et al., 2020), we found them only among right wingers, in line with Frei and Nachtwey's (2021) (see also Nachtwey et al., 2020) study about Corona protesters. However, our results go one step further by additionally demonstrating that left-wingers are systematically *less* prone to considering Corona a hoax than centrists. Another striking finding is the strong nexus between low income and Corona denialism (see also Schließler et al., 2020). So far, the role of financial deprivation seems to be underestimated as a motif to adopt Corona skepticism—especially in East Germany.

Third, our panel data enabled us to unearth differences in the socio-political profiles of former, new, and consistent Corona deniers. Most importantly, the latter group is the only group for whom we find an unequivocal association with right-wing self-identification, low education, and low income. These associations suggest that feelings of socioeconomic deprivation and a lack of social recognition may motivate this group, a presumption that could be examined in upcoming studies. A bit surprisingly, the group of new deniers does not differ much from the majority population in terms of the basic socio-political profile, except that they are younger and over-proportionally from the East (as Corona skeptics generally). What the new deniers and the consistent deniers unites is their strong preference for social media; quite obviously, the emergence of closed communication bubbles of like-minded poses a problem for social cohesion.

Fourth, we could confirm that various attitudes and dispositions are associated with supporting Corona skepticism, among them institutional distrust, trust in social media, political priorities (freedom rather than health), belief in specific Corona myths, and the value orientation of anti-conformity (yet not anti-universalism, as Spöri and Eichhorn, 2021 had suggested). While these findings largely support previous studies (cf. van Mulukom et al., 2022), a new finding is that the three types of former, new, and consistent corona deniers differ in their attitudinal profile: By and large, the viewpoints of the consistent deniers are the most extreme, followed by new deniers, and former deniers. Thus, the group of consistent deniers is most problematic from the perspective of social cohesion, as this group's mindset is most distant from that of the large majority.

Finally, our analysis provides valuable insights into the much-discussed East-West differences of Corona denialism in Germany. The idea that Corona is nothing but a hoax is considerably more common in the Eastern part—there, especially low-income earners and unpartnered hold this view. In contrast, political ideology (left-right self-placement) is less important for Corona denialism in the East, mainly because this view extents way into the camp of the centrists. The attitudinal profile of skeptics, however, is quite similar in East and West Germany, including for the group of consistent deniers. Put differently, Corona deniers in the East are not more extreme in their attitudes than their counterparts in the West. These findings may contribute to understanding why anti-Corona protests have been more widespread in Germany's Eastern part (though by no means confined to it): it is a matter of the *size* of the camp of Corona deniers, not a matter of its attitudinal profile. In addition, with the right-wing party AfD (Alternative for Germany/Alternative für Deutschland), which is more firmly anchored in the East, and with the anti-migration movement PEGIDA there was a denser network of political entrepreneurs in the East to mobilize Corona skeptics.

It goes without saying that our study is not without limitations. While the overall sample is of high quality and decent size, the sub-group sample sizes are limited, especially for the smallest group, the new Corona skeptics. Therefore, we may be missing certain associations that would reveal themselves as significant if the sample sizes had been larger (this may also hold for the East-West comparison). To avoid small case numbers, we could not always differentiate effects in as nuanced a way as may have been desirable. In terms of personal pandemic affectedness, for example, one might see differences in Corona denialism between those more marginally affected (e.g., becoming ill with mild symptoms) and those heavily affected (e.g., experiencing COVID-19-related deaths in the family), yet we had to collapse this information in our analysis.

Moreover, the operationalizations used for key variables are based on the public discourse at the very beginning of the pandemic. To make use of the panel structure, the item wording chosen in the first survey had to stay consistent in later waves. That, however, resulted in some wordings not being as closely aligned with how public discourses developed later. For example, a separation of the COVID-19 hoax item in denialism and disapproval of government action would have added more nuances. While the present survey allowed us to cover a wide range of determinants, it could not address everything that may be associated with conspiracy beliefs. Therefore, next to triangulating our results with other quantitative studies, qualitative work could give deeper insights into what motivates people to support Corona skepticism.

## Data availability statement

The raw data supporting the conclusions of this article will be made available by the authors, without undue reservation.

## Author contributions

JE took the lead in writing the paper. TS conducted the empirical analyses and prepared the model structure. JD contributed to conceptualization and analysis and structuring of the write-up. FD co-developed the methodology and data and contributed to the analysis. GD developed the variable operationalization and full regression development and contributed to the writing. All authors contributed to the article and approved the submitted version.

## Funding

The data collection and research is part of the project Values in Crisis: A Crisis of Values? Moral Values and Social Orientations under the Imprint of the Corona Pandemic, funded by Volkswagen Foundation, Grant No. 99/127.

## Conflict of interest

Author TS was employed by d|part. Author JE is a non-salaried director of d|part. The remaining authors declare that the research was conducted in the absence of any commercial or financial relationships that could be construed as a potential conflict of interest.

## Publisher's note

All claims expressed in this article are solely those of the authors and do not necessarily represent those of their affiliated organizations, or those of the publisher, the editors and the reviewers. Any product that may be evaluated in this article, or claim that may be made by its manufacturer, is not guaranteed or endorsed by the publisher.

## Footnotes

^1^The categorization of respondents to income classes is based on their equivalized net household income. Respondents were asked to report their net household income along 16 income brackets – weekly, monthly or yearly, as they found it more convenient. The corresponding monthly brackets were set to the average amount within a respective bracket (the lowest bracket to its upper bound, the highest bracket to its lower bound). The so-computed income of each respondent was equivalized by applying the square root of the reported household size. Adapting the approach of Krause et al. (2017) to the empirical distribution of the resulting equivalized monthly net household income, we defined the five income classes used in our analyses as follows (with respect to the sample median): low (up to 60 %), lower-middle (above 60% up to 100%), middle (above 100 % up to 130 %), upper-middle (above 130 % up to 169 %), high (above 169%).

^2^Respondents' political views were measured with the item: “In political matters, people talk of “the left” and “the right.” How would you place your views on this scale, generally speaking?”. The answering options ranged from 1 (left) to 10 (right). Respondents who selected 1 to 4 were categorized as identifying with the ‘left-wing’, 5 or 6 with the ‘centre’, 7 to 10 with the ‘right-wing’.

^3^Respondents were asked a number of yes-no questions on their health situation in relation to COVID-19: “I have been tested positively”, “I have or had mild symptoms”, “I have or had severe symptoms”, “People close to me have or had mild symptoms”, “People close to me have or had severe symptoms”, “People close to me have died as a result of an infection”. If a respondent answered “Yes” to at least one of these questions, they are considered as having been affected by COVID-19.
