# Supplementary material for: Reality bites: An analysis of corona deniers in Germany over time
Source: Front Sociol. 2022 Nov 4;7:974972. doi: 10.3389/fsoc.2022.974972 (PMC9672379; doi:10.3389/fsoc.2022.974972)
Supplement: Supplementary file 1 [file Data_Sheet_1.docx]

**Appendix**

**Table A1: Descriptive statistics for variables in analyses**

|  | **Minimum** | **Mean** | ***SD*** | **Maximum** |
| --- | --- | --- | --- | --- |
| *Dependent variables* |  |  |  |  |
| Denial of Corona | 0 | 0.095 | d | 1 |
| Type of Corona denier |  |  |  |  |
| Consistent realist | 0 | 0.831 | d | 1 |
| Former denier | 0 | 0.074 | d | 1 |
| New denier | 0 | 0.034 | d | 1 |
| Consistent denier | 0 | 0.060 | d | 1 |
| *Socio-political characteristics* |  |  |  |  |
| Sex: male (vs. female) | 0 | 0.507 | d | 1 |
| Age group |  |  |  |  |
| 16-34 years | 0 | 0.184 | d | 1 |
| 35-64 years | 0 | 0.427 | d | 1 |
| 65+ years | 0 | 0.388 | d | 1 |
| Partner: yes (vs. no) | 0 | 0.584 | d | 1 |
| Children: no (vs. no) | 0 | 0.573 | d | 1 |
| Education |  |  |  |  |
| low | 0 | 0.211 | d | 1 |
| intermediate | 0 | 0.551 | d | 1 |
| high | 0 | 0.238 | d | 1 |
| Income class |  |  |  |  |
| low | 0 | 0.217 | d | 1 |
| lower-middle | 0 | 0.298 | d | 1 |
| middle | 0 | 0.141 | d | 1 |
| upper-middle | 0 | 0.241 | d | 1 |
| high | 0 | 0.102 | d | 1 |
| Settlement |  |  |  |  |
| city or suburb | 0 | 0.427 | d | 1 |
| town | 0 | 0.353 | d | 1 |
| village | 0 | 0.220 | d | 1 |
| Germany: East (vs. West) | 0 | 0.173 | d | 1 |
| Political views |  |  |  |  |
| left-wing | 0 | 0.338 | d | 1 |
| center | 0 | 0.501 | d | 1 |
| right-wing | 0 | 0.162 | d | 1 |
| COVID-19 affected | 0 | 0.323 | d | 1 |
| *Attitudes and dispositions* |  |  |  |  |
| Institutional distrust | 1 | 2.309 | 0.635 | 4 |
| Social over traditional media | 1 | 2.063 | 1.004 | 5 |
| Conformity | 1 | 2.491 | 1.114 | 4 |
| Universalism | 1 | 2.458 | 1.100 | 4 |
| Freedom over health | 1 | 2.298 | 1.160 | 5 |
| Corona myths | 1 | 2.139 | 1.159 | 5 |

Notes. The table shows descriptive statistics (minimum, mean, *SD*: standard deviation, maximum) for the variables involved in the analysis. d: dummy variable. *N* = 1,280.

**Table A2: One-way ANOVA of attitudes by type of Corona deniers**

| **Variable** | **Type of Corona denier** | **Mean** | **Pairwise comparisons** | | | | **Overall ANOVA test** | |
| --- | --- | --- | --- | --- | --- | --- | --- | --- |
|  |  |  | **vs.** | **Mean difference** | ***SE*** |  | ***F*** |  |
| Distrust in institutions | Former | 2.749 | – Consistent | -.334 | .10 | *** | 7.40 | *** |
|  | New | 2.723 | – Former | -.027 | .11 |  |  |  |
|  | Consistent | 3.083 | – New | .360 | .12 | ** |  |  |
|  |  |  |  |  |  |  |  |  |
| Trust in social media | Former | 2.495 | – Consistent | -.674 | .15 | *** | 11.27 | *** |
|  | New | 3.023 | – Former | .528 | .18 | ** |  |  |
|  | Consistent | 3.169 | – New | .146 | .18 |  |  |  |
|  |  |  |  |  |  |  |  |  |
| Conformity | Former | 2.105 | – Consistent | .235 | .16 |  | 5.61 | *** |
|  | New | 2.545 | – Former | .440 | .19 | * |  |  |
|  | Consistent | 1.870 | – New | -.675 | .20 | *** |  |  |
|  |  |  |  |  |  |  |  |  |
| Universalism | Former | 2.158 | – Consistent | .171 | .17 |  | 0.56 |  |
|  | New | 2.136 | – Former | -.022 | .20 |  |  |  |
|  | Consistent | 1.987 | – New | -.149 | .21 |  |  |  |
|  |  |  |  |  |  |  |  |  |
| Freedom | Former | 2.884 | – Consistent | -.778 | .16 | *** | 11.16 | *** |
|  | New | 3.205 | – Former | .320 | .20 |  |  |  |
|  | Consistent | 3.662 | – New | .458 | .20 | * |  |  |
|  |  |  |  |  |  |  |  |  |
| Corona myths | Former | 2.835 | – Consistent | -.849 | .16 | *** | 14.12 | *** |
|  | New | 3.295 | – Former | .460 | .19 | * |  |  |
|  | Consistent | 3.684 | – New | .389 | .20 |  |  |  |

Notes. The tables shows the results from a one-way analysis of variance of each attitude/disposition as compared across the types of Corona deniers. For all *F*-scores reported, *df_1_* = 2, *df_2_* = 213. *N* = 216. The pairwise comparisons employ the Scheffé test. *SE* = standard error of the mean difference.
Significance of the estimates in two-sided tests: * *p* ≤ 0.10, ** *p* ≤ 0.05, *** *p* ≤ 0.01.
